# CD36 Gene Polymorphisms Are Associated with Intracerebral Hemorrhage Susceptibility in a Han Chinese Population

**DOI:** 10.1155/2017/5352071

**Published:** 2017-07-19

**Authors:** Qiu-Wen Gong, Mao-Fan Liao, Liang Liu, Xiao-Yi Xiong, Qin Zhang, Qi Zhong, Kai Zhou, Yuan-Rui Yang, Zhao-You Meng, Chang-Xiong Gong, Rui Xu, Qing-Wu Yang

**Affiliations:** Department of Neurology, Xinqiao Hospital, The Third Military Medical University, No. 183, Xinqiao Main Street, Shapingba District, Chongqing 400037, China

## Abstract

The CD36 gene encodes a membrane glycoprotein (type B scavenger receptor, SR-B2) that plays a crucial role in lipid sensing, innate immunity, atherogenesis, and glycolipid metabolism. In this study, we aimed to investigate the association between CD36 gene polymorphisms and intracerebral hemorrhage (ICH) in a Han Chinese population. We performed genotype and allele analyses for eleven single nucleotide polymorphisms (SNPs) of CD36 in a case-controlled study involving 292 ICH patients and 298 control participants. Eleven SNPs were genotyped by the Improved Multiple Ligase Detection Reaction (iMLDR) method. The results indicated that the SNP rs1194182 values were significantly different between ICH group and control group in a dominant model after adjusting for confounding factors. The subgroup analysis conducted for rs1194182 showed that the allele G frequencies were significantly different between ICH patients and controls in hypertension group via a dominant model. We then analyzed the rs1194182 genotype distributions among different groups of the serum lipid groups, including BMI, TC, TG, HDL, and LDL. However, no significant differences were found in the analysis of other subgroups. Taken together, these findings indicate that rs1194182 polymorphism in the CD36 gene was associated with ICH, and genotype GG could be an independent predictor.

## 1. Introduction

Intracerebral hemorrhage (ICH) is a crucial classification of stroke and has a high rate of mortality and morbidity [1, 2]. The pathogenesis of ICH is complex and influenced by environmental factors and genetic factors. However, its pathogenesis is not yet clear. Hypertension is the most common contributing factor to primary intracerebral hemorrhage. Factors including smoking, drinking, diabetes, and male gender increase the risk further [3–5]. In addition, many studies have shown an inverse association between the level of serum cholesterol and ICH [6]. More importantly, the role of genetics in the pathogenesis of ICH has received wide attention [7, 8]. Epidemiological studies have demonstrated that a history of a first-degree relative with ICH is an independent risk factor for lobar and nonlobar ICH [4]. It has also been reported that a family history of any stroke was a significant risk factor for patients with ICH who were <70 years compared with those who were >70 years [9]. Taken together, the observed differences based on family history indicate that genetic factors play an important role in the incidence of ICH.

CD36 is a type B scavenger receptor, now officially designated as SR-B2, and CD36 gene located in the 7q11.2 chromosome with 17 exons and 16 introns and is expressed on the surface of various cells: platelets, microvascular endothelial cells, monocytes/macrophages, dendritic cells, adipocytes, striated muscle cells, and hematopoietic cells [10, 11]. As a result of its expression in multiple cell types, it can be involved in a variety of biological processes, including transport of oxidized LDL (oxLDL) and fatty acids by macrophages and monocytes, and it participates in processes of inflammation, phagocytosis, and endocytosis [12, 13]. In addition, a wide variety of studies have investigated the important roles CD36 plays in many disorders, such as coronary heart disease, hypertension, Alzheimer's Disease, insulin resistance, and metabolic syndrome [14–17]. However, the relationship between CD36 gene polymorphisms and the risk of ICH has not yet been studied. Thus, to clarify the association of CD36 with ICH, we conducted this case-control study to find any SNPs of the CD36 gene associated with the risk of ICH.

## 2. Material and Methods

### 2.1. Ethics Statement

The study was approved by the local Ethics Committee of Xinqiao Hospital, Third Military Medical University (Chongqing, China), and written consent forms for genetic screening were obtained for all participants from the participant or from their legal representatives.

### 2.2. Study Population

In this study, a total of 292 patients with ICH were consecutively recruited from Chongqing Xinqiao Hospital from October 2014 to November 2016. All patients were diagnosed with ICH based on results of brain computed tomography (CT) scan and/or magnetic resonance imaging (MRI). The subjects were not eligible if the ICH was caused by trauma, neoplasms, anticoagulant therapy, coagulation disorders, aneurysms, or vascular malformations or if the patient declined to participate in this study. The 298 participants in the control group were randomly selected from the health examination center of Chongqing Xinqiao Hospital during the same period. The inclusion criterion for the controls was the absence of symptoms or medical history of stroke. The baseline characteristics and vascular risk factors were recorded, including age, gender, height, weight, body mass index (BMI), hypertension, coronary heart disease, diabetes mellitus, smoking and drinking habits, total cholesterol (TC), triglyceride (TG), high density lipoprotein cholesterol (HDL-c), low-density lipoprotein cholesterol (LDL-c), and family history of stroke, which are listed in Table 1. Smokers or drinkers were defined as participants who smoked ≥100 cigarettes or drank ≥12 times a year. BMI = weight/height^2^ (kg/m^2^).

### 2.3. Polymorphism Selection and Genotyping

SNPs were selected based on their functional relevance and minor allele frequency from Beijing (CHB) genotype data in the HapMap database (release #28, August 2010, https://www.hapmap.org/). The inclusion criteria were as follows: (1) all eligible SNP minor allele frequencies > 0.05 in the HapMap database, (2) SNP mutations led to amino acid changes according to the dbSNP, and (3) SNPs were located in one haplotype block and were in complete linkage disequilibrium (LD) (determined with the criterion of *r*^2^ > 0.8). Eleven common SNPs spanning the CD36 gene were included. Detailed information of each SNP is shown in Table 2.

Venous blood were collected in EDTA-coated vials after at least a 12-hour overnight fasting period. And the ICH patients' venous blood were collected within 24 h after ICH occurred. Genomic DNA was extracted using the Wizard Genomic DNA Purification Kit (Promega, USA) following the manufacturer's instructions. Improved Multiple Ligase Detection Reaction (iMLDR) was applied for genotyping [18]. Data analysis was carried out using GeneMapper Software version 4.0. Genotyping was carried out blind to group status.

### 2.4. Statistical Analysis

Statistical analyses were performed using SPSS 17.0 software (SPSS, Inc., Chicago, IL, USA). The presence of the Hardy-Weinberg equilibrium was determined using the chi-square test. The categorical variables were tested using the chi-squared (*χ*^2^) test. Categorical variables are expressed as proportions (%) and continuous variables as the mean ± standard deviation. For continuous variables, all comparisons between ICH group and the control group were made using independent *t*-test and among genotype groups by one-way ANOVA followed by LSD test and multiple comparisons. The genotype and allele distributions in ICH patients and control subjects were determined using the chi-squared (*χ*^2^) test. Multivariate logistic regression was used to analyze the genotype frequency of the subjects specified by different genetic models (additive, dominant and recessive comparison) and to calculate the *P* value, odds ratios (ORs), and 95% confidence intervals (CIs) after adjustment for covariates. Statistically significance was set at *P* < 0.05.

## 3. Results

A total of 292 ICH patients and 298 control subjects were included in this study. The clinical and laboratory parameters of all subjects in this study are presented in Table 1. The average age and sex distribution were similar between the ICH group and the control group (*P* > 0.05). As previous studies found, the prevalence of hypertension, drinking history, and family history in the ICH group were significantly higher than in the control group, and there were significant differences between the two groups (*P* < 0.05). BMI values and TC, TG, and LDL-C levels were also slightly raised in ICH patients. There were no significant differences between the two groups in gender, HDL, smoking history, and prevalence of coronary heart disease and diabetes mellitus.

Basic information of the eleven selected SNPs is shown in Table 2. The genotype and allelic frequencies of the SNPs in the ICH group and control group are shown in Table 3. All of the genotypes of eleven SNPs were in agreement with the HWE for the control group (*P* > 0.05), which indicates that the data remain constant in the population (data not shown).

Logistic regression analysis was performed after adjusting for age, gender, body mass index, hypertension, coronary heart disease, diabetes mellitus, smoking and drinking habits, TC, TG, HDL, LDL, and family history of stroke. A significant association between CD36 and ICH was seen in rs1194182, showing that genotype GG was a risk factor for ICH compared with genotype GC-CC (dominant model, OR = 0.674, 95% CI 0.457–0.992, *P* = 0.046). However, allelic frequencies of rs1194182 in the ICH group and control group have no significant difference. In addition, no associations between the other ten SNPs and the risk of ICH were observed in this study.

All the participants were divided into subgroups to examine whether there were associations between CD36 and ICH in gender or hypertension, ICH location, which are listed in Table 4. After logistic regression analysis for rs1194182, the genotype GG distribution (dominant model, OR = 0.578, 95% CI 0.341–0.98, *P* = 0.042) and the allele G frequencies (OR = 1.533, 95% CI 1.095–2.145, *P* = 0.012) were significantly different between ICH patients and controls in the hypertension group. And among nonlobar ICH group, we found a significant difference in the genotype of GG distribution compared to controls (dominant model, OR = 0.645, 95% CI 0.461–0.997, *P* = 0.043). No significant association could be found in the normotensive group, nonlobar ICH group, the male group, and the female group. CD36 is involved in a variety of roles in lipid metabolism. We then analyzed the rs1194182 genotype distributions among different subgroups, including BMI, TC, TG, HDL, and LDL. However, no significant differences were found in different lipid groups (Table 5).

## 4. Discussion

In our study, we identified eleven SNPs of CD36 and found that SNP rs1194182 has significant differences in this case-controlled study among a Chinese Han population after adjusting for age, gender, body mass index, hypertension, coronary heart disease, diabetes mellitus, smoking and drinking habits, TC, TG, HDL, LDL, and family history of stroke. Subgroup analysis conducted for the SNP rs1194182 in CD36 showed that GG genotype frequencies were significantly different between ICH patients and controls in hypertension group via a dominant model, especially in the hypertension group.

ICH is an important subtype of stroke and is etiologically diverse. The pathogenesis of ICH involves many factors. As found in our study, CD36 SNP rs1194182 may increase the risk of ICH. However, the mechanism by which CD36 increases the risk of ICH is unknown. It is known that hypertension plays an important role in the pathogenesis of ICH [19], and many studies have found that CD36 is closely related to the development of hypertension [14, 20, 21]. In a microarray analysis study of the differential gene expression between hypertensives and normotensives, 31 genes were upregulated and 18 genes were downregulated, including the CD36 gene with 4.8-fold changes and significant differences between hypertensive and normotensive groups [22]. CD36 deficient individuals were found to have increased blood pressure levels [23]. The +273A/G polymorphism in CD36 was associated with essential hypertension especially in males [14]. Thus, CD36 may contribute to the development of ICH by the following mechanisms. One possibility is through regulating the function of endothelin-1 and altering the properties of vascular smooth muscle, which lead to the development of hypertension and atherosclerosis [24]. Pravenec et al. found that deficiency in renal expression of CD36 could increase blood pressure [21, 25]. In our study, the results showed that rs1194182 polymorphism in the CD36 was significantly associated with ICH in the hypertension group, which indicated that significant correlation existed between rs1194182 polymorphism and hypertension on ICH. Moreover, previous study found that ICH occurring in different location may have different pathophysiology, among all cases of lobar ICH, which mainly caused by an apolipoprotein E4 or E2 allele. But about half of all cases of nonlobar ICH are attributable to hypertension [4]. Thus, we assume that CD36 gives rise to ICH through blood pressure pathways. In addition, SNP rs1194182 in the CD36 gene could be a molecular marker for ICH particularly in hypertensive patients.

CD36 is involved in a variety of lipid metabolism pathways. CD36 has the ability to facilitate the uptake of long-chain fatty acids in muscle and adipose tissues, which contributes to the regulation of lipid metabolism and insulin resistance [26, 27]. CD36 is also expressed on macrophages as a receptor for oxidized low-density lipoproteins and plays an important role in the development of atherosclerosis [28]. Previous Japanese studies have reported an association between rare CD36 variants and high blood levels of free fatty acid and triglycerides [29, 30]. CD36 sequence variants were also associated with HDL-C levels [31]. A meta-analysis found that the CD36 gene was significantly linked to triglycerides and triglycerides/HDL-C ratio but not linked to LDL or total cholesterol [32]. In addition, many studies have demonstrated that the polymorphism of CD36 influences the serum lipid levels in the patients of atherosclerosis, coronary heart disease, and metabolic syndrome [33–35]. Importantly, as the Rotterdam Study found, low serum triglycerides levels were also associated with an increased risk of ICH [36]. To evaluate the influence of those factors on the genotype of CD36 in this study, we conduct subgroups analysis according to the BMI, TC, TG, HDL, and LDL. However, for rs1194182, there was no significant difference among lipid subgroups found in the analysis. These findings may indicate that the CD36 increased risk of ICH is not through dyslipidemia pathways.

To our knowledge, this study was the first to report an association between SNPs of CD36 and ICH in a Chinese Han population. This association may be stronger for those individuals labeled as ICH. The SNP rs1194182 in CD36 can be recognized as molecular markers of ICH even though the mechanism is not clear. However, the main limitations of this study are the small sample size and the mechanisms of the CD36 gene in ICH are still unknown. These results should be validated in a large population and in different ethnicities. In our future studies, we will work on the mechanisms of the CD36 gene in ICH with a larger sample size in more diverse areas. As the association between SNPs of CD36 and ICH has been confirmed in a larger sample size and more diverse areas, we would estimate individual risk of ICH just according the venous blood sample.

## Figures and Tables

**Table 1:** Demographic and clinical characteristics of ICH patients and controls.

Characteristics	Patients (*n* = 292)	Controls (*n* = 298)	*P* value
Age (years ± SD)	57.12 ± 12.52	58.84 ± 13.61	0.112
Gender, (M/F)	149 (143)	151 (147)	0.931
BMI (kg/m^2^)	24.06 ± 3.46	23.44 ± 3.43	0.029
Hypertension, *n* (%)	227 (77.74)	108 (36.24)	0
Coronary heart disease	27 (265)	21 (238)	0.231
Diabetes mellitus	36 (256)	40 (258)	0.692
Smoking history	69 (223)	59 (239)	0.259
Drinking history	47 (245)	31 (267)	0.041
Family history	12 (280)	4 (294)	0.039
TC (mmol/L)	4.65 ± 1.05	4.10 ± 1.05	0
TG (mmol/L)	2.23 ± 5.41	1.58 ± 1.4	0.049
HDL-c (mmol/L)	1.14 ± 0.33	1.13 ± 0.38	0.712
LDL-c (mmol/L)	3.03 ± 0.76	2.73 ± 0.88	0

**Table 2:** Characteristics of CD36 gene polymorphisms investigated in the study.

SNPs	Chromosome	Position	SNP property	Length	Alleles	MAF (CHB_1000 g)
rs1049654	7	80275455	5′UTR	379	A>C	0.325
rs1049673	7	80306350	3′UTR	308	C>G	0.485
rs1194182	7	80231504	5′UTR	256	G>C	0.335
rs12666644	7	80308199	3′UTR	279	G>A	0.087
rs12706949	7	80307224	3′UTR	354	G>T	0.427
rs12706950	7	80307502	3′UTR	244	G>A	0.422
rs13223096	7	80307624	3′UTR	244	C>T	0.427
rs13226433	7	80308171	3′UTR	279	C>A	0.485
rs13246513	7	80306751	3′UTR	250	C>T	0.422
rs1334512	7	80267904	5′UTR	288	G>T	0.272
rs7755	7	80306271	3′UTR	308	G>A	0.485

**Table 3:** Comparison of the genotype and allele frequencies of associated CD36 gene variants between ICH patients and controls.

SNPs	Genotype	Frequency		Additive model		Dominant model		Recessive model		Allele	Frequency		Multiplicative model	
		ICH patients, *n* 292(%)	Control patients, *n* 298(%)	*P*	OR (95% CI)	*P*	OR (95% CI)	*P*	OR (95% CI)		ICH patients, *n* (%)	Control patients, *n* (%)	*P*	OR (95% CI)
rs1049654	AA	135 (46.2)	115 (38.6)	0.112	0.798 (0.604–1.054)	0.069	0.698 (0.474–1.028)	0.569	0.849 (0.484–1.49)	A	394 (67.5)	373 (62.6)	0.079	1.24 (0.975–1.576)
	AC	124 (42.5)	143 (48.0)							C	190 (32.5)	223 (37.4)		
	CC	33 (11.3)	40 (13.4)											

rs1049673	CC	60 (20.5)	63 (21.2)	0.847	1.027 (0.783–1.346)	0.894	0.972 (0.638–1.48)	0.632	1.121 (0.703–1.787)	C	265 (45.4)	276 (46.3)	0.748	0.963 (0.766–1.211)
	CG	145 (49.7)	150 (50.3)							G	319 (54.6)	320 (53.7)		
	GG	87 (29.8)	85 (28.5)											

rs1194182	GG	137 (46.9)	118 (39.6)	0.072	0.772 (0.583–1.024)	**0.046**	**0.674 (0.457**–**0.992)**	0.481	0.814 (0.459–1.445)	G	398 (68.2)	378 (63.4)	0.087	0.81 (0.637–1.031)
	GC	124 (42.5)	142 (47.7)							C	186 (31.8)	218 (36.6)		
	CC	31 (10.6)	38 (12.7)											

rs12666644	GG	258 (88.3)	245 (82.2)	0.208	0.723 (0.436–1.198)	0.131	0.664 (0.39–1.13)	0.381	3.211 (0.237–43.56)	G	548 (93.8)	542 (90.9)	0.979	1.517 (0.979–2.35)
	GA	32 (11.0)	52 (17.5)							A	36 (6.2)	54 (9.1)		
	AA	2 (0.7)	1 (0.3)											

rs12706949	GG	83 (28.4)	88 (29.5)	0.787	0.963 (0.734–1.264)	0.509	0.868 (0.57–1.322)	0.786	1.067 (0.667–1.707)	G	315 (53.9)	323 (54.2)	0.93	0.99 (0.787–1.244)
	GT	149 (51.0)	147 (49.3)							T	269 (46.1)	273 (45.8)		
	TT	60 (20.6)	63 (21.2)											

rs12706950	GG	82 (28.1)	88 (29.5)	0.788	0.963 (0.734–1.264)	0.531	0.874 (0.574–1.332)	0.814	1.058 (0.662–1.69)	G	314 (53.8)	322 (54.0)	0.929	0.99 (0.787–1.244)
	GA	150 (51.4)	146 (49.0)							A	270 (46.2)	274 (46.0)		
	AA	60 (20.5)	64 (21.5)											

rs13223096	CC	83 (28.4)	88 (29.5)	0.787	0.963 (0.734–1.264)	0.509	0.868 (0.57–1.332)	0.786	1.067 (0.667–1.707)	C	315 (53.9)	323 (54.2)	0.93	0.99 (0.787–1.244)
	CT	149 (51.0)	147 (49.3)							T	269 (46.1)	273 (45.8)		
	TT	60 (20.6)	63 (21.2)											

rs13226433	CC	59 (20.2)	62 (20.8)	0.833	1.03 (0.785–1.351)	0.869	0.965 (0.634–1.469)	0.584	1.14 (0.713–1.825)	C	264 (45.2)	275 (46.1)	0.747	0.963 (0.766–1.211)
	CA	146 (50.0)	151 (50.7)							A	320 (54.8)	321 (53.9)		
	AA	87 (29.8)	85 (28.5)											

rs13246513	CC	83 (28.4)	89 (29.9)	0.798	0.965 (0.735–1.267)	0.568	0.885 (0.528–1.346)	0.482	1.049 (0.655–1.68)	C	316 (54.1)	324 (54.4)	0.931	0.99 (0.787–1.245)
	CT	150 (51.4)	146 (49.0)							T	268 (45.9)	272 (45.6)		
	TT	59 (20.2)	63 (21.1)											

rs13344512	GG	172 (58.9)	188 (63.1)	0.11	1.302 (0.942–1.8)	0.246	1.259 (0.583–1.859)	0.09	2.203 (0.884–5.487)	G	441 (75.5)	477 (80.0)	0.062	0.769 (0.584–1.013)
	GT	97 (33.2)	101 (33.9)							C	143 (24.5)	119 (20.0)		
	TT	23 (7.9)	9 (3.0)											

rs7755	GG	60 (20.6)	63 (21.2)	0.922	1.014 (0.773–1.328)	0.78	0.942 (0.619–1.433)	0.632	1.121 (0.703–1.787)	G	264 (45.2)	276 (46.3)	0.704	0.957 (0.761–1.203)
	GA	144 (49.3)	150 (50.3)							A	320 (54.8)	320 (53.7)		
	AA	88 (30.1)	85 (28.5)											

CI, confidence interval; OR, odds ratio; SNP, single nucleotide polymorphism. Additive model: heterozygotes and minor allele homozygotes were weighed 2 and 1, respectively, to major allele homozygotes. Dominant model: major allele homozygotes versus heterozygotes plus minor allele homozygotes. Recessive model: major allele homozygotes plus heterozygotes versus minor allele homozygotes.

**Table 4:** The genotype distributions and allele frequencies of the CD36 gene rs1194182 polymorphisms in the hypertension and normotensive groups.

Subgroup	rs1194182			Additive model		Dominant model		Recessive model		Allele		Multiplicative model	
	GG	GC	CC	*P*	OR (95% CI)	*P*	OR (95% CI)	*P*	OR (95% CI)	G	C	*P*	OR (95% CI)
Male (ICH)(149)	69	64	16	0.613	0.905 (0.161–1.331)	0.601	0.866 (0.505–1.485)	0.79	0.9 (0.415–1.95)	202	96	0.242	1.223 (0.873–1.713)
Male (control)(151)	62	67	22							191	111		
Female (ICH)(143)	68	60	15	0.601	0.682 (0.33–1.409)	0.201	0.498 (0.171–1.45)	0.791	0.83 (0.209–3.293)	196	90	0.21	1.246 (0.883–1.759)
Female (control)(147)	56	75	16							187	107		
Hypertension (ICH)(227)	105	100	22	0.053	0.696 (0.482–1.005)	**0.042**	**0.578 (0.341**–**0.98)**	0.333	0.7 (0.334–1.441)	310	144	**0.012**	**1.533 (1.095**–**2.145)**
Hypertension (control)(107)	36	53	18							125	89		
Normotensive (ICH)(65)	32	24	9	0.982	1.005 (0.64–1.58)	0.539	0.828 (0.453–1.513)	0.307	1.602 (0.648–3.96)	88	42	0.76	1.068 (0.699–1.633)
Normotensive (control)(191)	82	89	20							253	129		
Lobar (ICH)(40)	18	19	3	0.973	1.018 (0.761–1.631)	0.604	0.947 (0.622–1.846)	0.423	1.098 (0.874–2.96)	55	25	0.351	1.269 (0.769–2.095)
Lobar (control)(298)	118	142	38							378	218		
Nonlobar (ICH)(252)	119	105	28	0.063	0.778 (0.311–1.592)	**0.043**	**0.645 (0.461**–**0.997)**	0.512	0.797 (0.384–1.524)	343	161	0.107	1.229 (0.956–1.579)
Nonlobar (control)(298)	118	142	38							378	218		

**Table 5:** The BMI and serum lipid data of ICH patients and control subjects stratified by CD36 genotypes.

Parameter	Genotype			*P* value (multiple comparison)
	GG	GC	CC	
BMI (ICH)	24.043 ± 3.423	24.311 ± 3.563	23.651 ± 4.042	0.59
BMI (control)	23.367 ± 3.053	23.479 ± 3.602	23.533 ± 3.916	0.952
TC (ICH)	4.7045 ± 1.112	4.649 ± 1.041	4.394 ± 0.748	0.332
TC (control)	4.117 ± 1.154	4.134 ± 0.927	3.908 ± 1.173	0.489
TG (ICH)	1.926 ± 1.671	1.927 ± 1.223	1.886 ± 1.109	0.989
TG (control)	1.64 ± 1.52	1.609 ± 1.413	1.292 ± 0.88	0.391
HDL (ICH)	1.174 ± 0.338	1.111 ± 0.316	1.149 ± 0.303	0.295
HDL (control)	1.113 ± 0.295	1.152 ± 0.451	1.131 ± 0.317	0.711
LDL (ICH)	3.076 ± 0.768	3.037 ± 0.794	2.841 ± 0.61	0.303
LDL (control)	2.739 ± 0.843	2.728 ± 0.919	2.627 ± 0.841	0.717

## References

[1] Murray C. J. L., Lopez A. D. (1997). Mortality by cause for eight regions of the world: global burden of disease study. *The Lancet*.

[2] Guzik A., Bushnell C. (2017). Stroke epidemiology and risk factor management. *CONTINUUM: Lifelong Learning in Neurology*.

[3] Kurth T., Kase C. S., Berger K., Gaziano J. M., Cook N. R., Buring J. E. (2003). Smoking and risk of hemorrhagic stroke in women. *Stroke*.

[4] Woo D., Sauerbeck L. R., Kissela B. M. (2002). Genetic and environmental risk factors for intracerebral hemorrhage: preliminary results of a population-based study. *Stroke*.

[5] Ariesen M. J., Claus S. P., Rinkel G. J. E., Algra A. (2003). Risk factors for intracerebral hemorrhage in the general population: a systematic review. *Stroke*.

[6] Wu J., Chen S., Zhou Y. (2013). Non-high-density lipoprotein cholesterol on the risks of stroke: a result from the Kailuan study. *PLoS ONE*.

[7] Anderson C. D., Falcone G. J., Phuah C. L. (2016). Genetic variants in CETP increase risk of intracerebral hemorrhage. *Annals of Neurology*.

[8] Carpenter A. M., Singh I. P., Gandhi C. D., Prestigiacomo C. J. (2016). Genetic risk factors for spontaneous intracerebral haemorrhage. *Nature Reviews Neurology*.

[9] An P., Rice T., Gagnon J. (2000). Familial aggregation of stroke volume and cardiac output during submaximal exercise: The HERITAGE family study. *International Journal of Sports Medicine*.

[10] Nicholson A. C., Han J., Febbraio M., Silversterin R. L., Hajjar D. P. (2001). Role of CD36, the macrophage class B scavenger receptor, in atherosclerosis. *Annals of the New York Academy of Sciences*.

[11] Febbraio M., Hajjar D. P., Silverstein R. L. (2001). CD36: a class B scavenger receptor involved in angiogenesis, atherosclerosis, inflammation, and lipid metabolism. *Journal of Clinical Investigation*.

[12] Young M. P., Febbraio M., Silverstein R. L. (2009). CD36 modulates migration of mouse and human macrophages in response to oxidized LDL and may contribute to macrophage trapping in the arterial intima. *The Journal of Clinical Investigation*.

[13] Rać M. E., Safranow K., Poncyljusz W. (2007). Molecular basis of human CD36 gene mutations. *Molecular Medicine*.

[14] Liu X., Meng F., Yang P. (2013). Association study of CD36 single nucleotide polymorphisms with essential hypertension in the Northeastern Han Chinese. *Gene*.

[15] Coraci I. S., Husemann J., Berman J. W. (2002). CD36, a class B scavenger receptor, is expressed on microglia in Alzheimer's disease brains and can mediate production of reactive oxygen species in response to *β*-amyloid fibrils. *The American Journal of Pathology*.

[16] Che J. J., Shao Y. X., Li G. P. (2014). Association between rs1049673 polymorphism in CD36 and premature coronary heart disease. *Genetics and Molecular Research*.

[17] Lu Z., Li Y., Brinson C., Kirkwood K., Lopes-Virella M., Huang Y. (2017). CD36 is upregulated in mice with periodontitis and metabolic syndrome and involved in macrophage gene upregulation by palmitate. *Oral Diseases*.

[18] Thomas G., Sinville R., Sutton S. (2004). Capillary and microelectrophoretic separations of ligase detection reaction products produced from low-abundant point mutations in genomic DNA. *Electrophoresis*.

[19] Nechikkat S., Chandni R., Sasidharan P. K. (2014). Hypertension as a risk factor for haemorrhagic stroke in females. *Journal of Association of Physicians of India*.

[20] Pravenec M., Kurtz T. W. (2002). Genetics of Cd36 and the hypertension metabolic syndrome. *Seminars in Nephrology*.

[21] Pravenec M., Churchill P. C., Churchill M. C. (2008). Identification of renal Cd36 as a determinant of blood pressure and risk for hypertension. *Nature Genetics*.

[22] Korkor M. T., Meng F. B., Xing S. Y. (2011). Microarray analysis of differential gene expression profile in peripheral blood cells of patients with human essential hypertension. *International Journal of Medical Sciences*.

[23] Miyaoka K., Kuwasako T., Hirano K.-I., Nozaki S., Yamashita S., Matsuzawa Y. (2001). CD36 deficiency associated with insulin resistance. *Lancet*.

[24] Kwok C. F., Juan C.-C., Ho L.-T. (2007). Endothelin-1 decreases CD36 protein expression in vascular smooth muscle cells. *American Journal of Physiology - Endocrinology and Metabolism*.

[25] Pravenec M., Kurtz T. W. (2007). Molecular genetics of experimental hypertension and the metabolic syndrome: from gene pathways to new therapies. *Hypertension*.

[26] (2017). CD36-mediated lipid metabolism promotes metastasis. *Cancer Discovery*.

[27] Glatz J. F. C., Angin Y., Steinbusch L. K. M., Schwenk R. W., Luiken J. J. F. P. (2013). CD36 as a target to prevent cardiac lipotoxicity and insulin resistance. *Prostaglandins Leukotrienes and Essential Fatty Acids*.

[28] Park Y. M. (2014). CD36, a scavenger receptor implicated in atherosclerosis. *Experimental and Molecular Medicine*.

[29] Furuhashi M., Ura N., Nakata T., Shimamoto K. (2003). Insulin sensitivity and lipid metabolism in human CD36 deficiency. *Diabetes Care*.

[30] Kajihara S., Hisatomi A., Ogawa Y. (2001). Association of the Pro90Ser CD36 mutation with elevated free fatty acid concentrations but not with insulin resistance syndrome in Japanese. *Clinica Chimica Acta*.

[31] Love-Gregory L., Sherva R., Sun L. (2008). Variants in the CD36 gene associate with the metabolic syndrome and high-density lipoprotein cholesterol. *Human Molecular Genetics*.

[32] Malhotra A., Elbein S. C., Ng M. C. Y. (2007). Meta-analysis of genome-wide linkage studies of quantitative lipid traits in families ascertained for type 2 diabetes. *Diabetes*.

[33] Yazgan B., Ustunsoy S., Karademir B., Kartal-Ozer N. (2014). CD36 as a biomarker of atherosclerosis. *Free Radical Biology and Medicine*.

[34] Baba M. I., Kaul D., Grover A. (2006). Importance of blood cellular genomic profile in coronary heart disease. *Journal of Biomedical Science*.

[35] Hoang-Yen Tran D., Hoang-Ngoc Tran D., Mattai S. A. (2016). Cathelicidin suppresses lipid accumulation and hepatic steatosis by inhibition of the CD36 receptor. *International Journal of Obesity*.

[36] Wieberdink R. G., Poels M. M. F., Vernooij M. W. (2011). Serum lipid levels and the risk of intracerebral hemorrhage: The Rotterdam study. *Arteriosclerosis, Thrombosis, and Vascular Biology*.

